# A machine-learning approach to predict postprandial hypoglycemia

**DOI:** 10.1186/s12911-019-0943-4

**Published:** 2019-11-06

**Authors:** Wonju Seo, You-Bin Lee, Seunghyun Lee, Sang-Man Jin, Sung-Min Park

**Affiliations:** 1 Department of Creative IT engineering, POSTECH, 77, Cheongam-Ro, Nam-Gu, Pohang, 37673 Republic of Korea; 2 Division of Endocrinology and Metabolism, Department of Medicine, Samsung Medical Center, Sungkyunkwan University School of Medicine, 81, Irwon-ro, Seoul, 06351 Republic of Korea

**Keywords:** Hypoglycemia, Risk prediction, Machine-learning approach, Diabetes

## Abstract

**Background:**

For an effective artificial pancreas (AP) system and an improved therapeutic intervention with continuous glucose monitoring (CGM), predicting the occurrence of hypoglycemia accurately is very important. While there have been many studies reporting successful algorithms for predicting nocturnal hypoglycemia, predicting postprandial hypoglycemia still remains a challenge due to extreme glucose fluctuations that occur around mealtimes. The goal of this study is to evaluate the feasibility of easy-to-use, computationally efficient machine-learning algorithm to predict postprandial hypoglycemia with a unique feature set.

**Methods:**

We use retrospective CGM datasets of 104 people who had experienced at least one hypoglycemia alert value during a three-day CGM session. The algorithms were developed based on four machine learning models with a unique data-driven feature set: a random forest (RF), a support vector machine using a linear function or a radial basis function, a K-nearest neighbor, and a logistic regression. With 5-fold cross-subject validation, the average performance of each model was calculated to compare and contrast their individual performance. The area under a receiver operating characteristic curve (AUC) and the F1 score were used as the main criterion for evaluating the performance.

**Results:**

In predicting a hypoglycemia alert value with a 30-min prediction horizon, the RF model showed the best performance with the average AUC of 0.966, the average sensitivity of 89.6%, the average specificity of 91.3%, and the average F1 score of 0.543. In addition, the RF showed the better predictive performance for postprandial hypoglycemic events than other models.

**Conclusion:**

In conclusion, we showed that machine-learning algorithms have potential in predicting postprandial hypoglycemia, and the RF model could be a better candidate for the further development of postprandial hypoglycemia prediction algorithm to advance the CGM technology and the AP technology further.

## Background

Intensive insulin treatment is a standard of care for tight glycemic control in people with diabetes, to prevent or delay long-term complications of diabetes mellitus [1–3] However, insulin therapy may cause life threatening hypoglycemia and thus achieving and maintaining near normoglycemia is largely limited by this risk factor [4], which persists despite advances in treatment technique [2–6]. Recently, artificial pancreas (AP) systems are emerging and they use machine-learning algorithms to reduce the frequency of hypoglycemic episodes, even in the presence of intensive insulin treatment, and are among the greatest advances in diabetes care in recent development [7, 8].

In the clinically approved hybrid AP system, mealtime insulin dosing depends on the carbohydrate counting by patients. This process requires extensive patient education and is a complex task to most patients, which can lead to entering inaccurate information and consequently miscalculation of insulin dosage [9]. For this reason, the single-hormone AP system delivers a reduced bolus insulin dose at mealtime, to avoid risk of postprandial hypoglycemia. The systems then reduce the post-meal hyperglycemia by increasing the infusion rate of basal insulin. This conservative dosing of mealtime bolus insulin explains in part why only modest daytime benefit has been achieved by the single-hormone AP system, in spite of impressive nocturnal glucose control. The recently proposed bi-hormonal AP system [6] controls both insulin and glucagon pump to reduce hypoglycemia while maintaining intensive insulin treatment, and thus does not require carbohydrate counting by patients. However, the use of an additional glucagon pump increases the system cost and complexity. Furthermore, the use of these systems can cause nausea, and raises long-term concerns about safety [10, 11]. It has been reported that single- and bi-hormonal artificial pancreas systems indeed control nocturnal glucose with equal effectiveness [12]. Despite the existence of algorithms that predict nocturnal hypoglycemia (for both single- and bi-hormonal AP systems) with high accuracy, the prediction of postprandial hypoglycemia is still a challenge because of extreme glucose fluctuations around mealtimes. Current rapid-acting insulin [13] analogues are still not equivalent to physiologic biphasic insulin secretion that precisely regulates the rapid changes in glucose intake, glucagon secretion, endogenous glucose production, and utilization of glucose around mealtimes[14]. This highlights the importance of developing an accurate and easy to use algorithm to predict postprandial hypoglycemia.

As a method for predicting a glucose level or the occurrence of the hypoglycemia, a physiological prediction method, a data-driven method, and a hybrid method were proposed [15, 16]. Among them, many data-driven models relying on continuous glucose monitoring (CGM) data and additional inputs such as diet, exercise, insulin injection dosage, and others have been proposed [15]. Firstly, there have been several studies to develop time series models [17–19] that can predict a glucose level. Sparacino et al. [17] proposed a first-order autoregressive (AR) and a first-order polynomial model to predict a glucose level with 30-min or 45-min prediction horizons. The proposed forgetting factor allowed the models to be more sensitive to the recent CGM data points, and thus the models were able to reflect rapid glucose changes around mealtimes. However, the models needed to tune their parameters at every sampling and the predicted glucose fluctuated significantly as the forgetting factor got smaller. An autoregressive integrated moving-average (ARIMA) model with an adaptive algorithm was recently proposed [18]. When the proposed model predicted hypoglycemia with the 30-min prediction horizon, time of detection was earlier and false alarm rate was lower than other models such as an adaptive univariate model and a general ARIMA model. However, this model requires large computations and is difficult to be implemented in real time. Another time series model with exogenous inputs such as meal information, insulin on board, and physical activity was recently proposed [19]. Although this model showed impressive performance for an early prediction of hypoglycemia, requirements for manual inputs makes this solution cumbrous, difficult to use and prone for errors [16].

Machine learning has made a great progress in medical data science and has been applied to a glucose level or hypoglycemia prediction as well [15, 16, 20–28]. The artificial neural network (ANN) proposed in [20] used five consecutive CGM data points and measurement time. Although ANN showed better predictive performance than time series model (e.g., AR model [17]), it could not detect sudden changes in a glucose level due to meals or insulin injections. However, this limitation could be overcome by inserting additional information such as meals [21–23] and insulin doses [23], but the cost of simplicity. A feed-forward neural network model has been presented in [24], but training this model also required burdensome inputs such as insulin dosage, nutritional intake, lifestyle, and emotional factor other than consecutive CGM data points. In addition to the neural network approach, there have been numerous efforts to predict hypoglycemia with an ensemble method [25], a decision-tree method [26, 27], and machine learning models using self-monitoring blood glucose (BG) data [28]. However, most of the related works required more than one form of manual inputs for more accurate prediction of hypoglycemia and did not focus on postprandial hypoglycemia except for our preliminary study [27].

The goal of this study is to develop an easy-to-use, computationally efficient machine-learning algorithm to predict postprandial hypoglycemia using unique data-driven features derived from the data. The glucose dynamics in daytime and nighttime are quite different due to meals and activities, and having a dedicated algorithm for the postprandial hypoglycemia would improve the AP’s insulin dosing during the daytime benefitting the patient significantly. In addition, computationally efficient algorithm, which does not require any parameter tuning on new dataset, with the reduced number of manual inputs will help the AP system to be more usable, compact, and clinically feasible. We validated postprandial hypoglycemia prediction with 30 min of the prediction horizon using retrospective CGM dataset of 104 patients described in the data “Data acquisition” section. As detailed in the “Methods” section, we used commonly used machine learning models to compare and contrast their individual performance, and finally determine the best candidate for the further development of the postprandial hypoglycemia prediction algorithm for the future AP system. In the next few subsections we present our work in greater detail.

## Methods

### Data acquisition

We reviewed the medical records of all 411 patients who underwent CGM at Samsung Medical Center (Seoul, Republic of Korea) between 2013 and 2015, with approval from the Institutional Review Board (IRB) of the Samsung Medical Center (IRB File number; SMC 2016-05-058-001). All medical procedures were performed in accordance with relevant guidelines and regulations. The informed consent requirement for this study was waived by the board because the researcher only accessed the database for analysis purposes, and all patient data were de-identified. From these records, we extracted 113 three-day CGM datasets from 110 Korean adults (aged ≥18 y) that met two inclusion criteria: 1) the CGM data contained at least one BG level ≤3.9 mmol/L (70 mg/dL); and 2) the primary purpose of performing CGM included evaluation of hypoglycemia. Of the 110 patients who met inclusion criteria, six were excluded: two patients had alimentary hypoglycemia after total gastrectomy, two patients took acetaminophen during the CGM, one patient had gestational diabetes and was not taking any anti-diabetic medications or insulin (n=1), and one patient had missing data. Of the remaining 104 patients (Table 1), 52 (aged 18-74 years) had type-1 diabetes mellitus and 52 (aged 32-80 years) had type-2 diabetes mellitus. During the study, none of the patients was under steroid therapy with doses higher than a prednisolone equivalent of 7.5 mg per day, and none was under cancer treatment such as chemotheraphy or radiotheraphy. One patient was undergoing treatment for diabetic foot infection. Three of the participants contributed two separate three-day CGM datasets separated by 9 to 22 months, so a total of 107 three-day CGM datasets were used.

**Table 1:** Clinical characteristics of enrolled study subjects

	Type-1 diabetes (55 three-day CGM datasets in 52 patients)	Type-2 diabetes (52 three-day CGM datasets in 52 patients)
Age (year)	40.0 (29.0-52.0)	63.5 (54.3-68.0)
Sex (male:female)	21:34*	21:31
Body weight (kg)	60.48 (52.35-69.41)	60.75 (54.60-70.37)
BMI (kg/ *m*^2^)	22.85 ±3.26	24.60 ±2.62
Duration of diabetes (years)	11.0 (6.0-18.0)	19.0 (13.3-25.0)
Insulin therapy (with insulin therapy: without insulin therapy)	55*:0	43:9
Insulin regimen basal:intermediate-acting: premix:MDI:CSII	3:1:6:44:1	20:3:11:9:0
Daily insulin dose (IU/day)	42.3 ±17.7	28.6 ±18.1
Daily insulin dose per body weight (IU/day/kg)	0.68 (0.53-0.82)	0.50 (0.30-0.60)
eGFR (ml/min/1.73 *m*^2^)	83.05 (71.98-96.95)	70.40 (51.30-82.50)
End stage renal disease [n (%)]	4 (7.3)	2 (3.8)
Liber cirrhosis [n (%)]	2 (3.6)	0 (0.0)
Heart failure with reduced ejection fraction [n (%)]	0 (0.0)	1 (1.9)
Pancreatic resection [n (%)]	2 (3.6)†	0 (0.0)
Acute infection [n (%)]	0 (0.0)	1 (1.9)
Pregnancy [n (%)]	1 (1.8)	0 (0.0)
Hemoglobin A1C (%)	7.94 ±1.13	8.31 ±1.32
C-peptide (ng/mL)	0.02 (0.02-0.15)	1.46 (0.80-2.44)

Continuous variables with normal distributions are expressed as mean ± standard deviation, whereas continuous variables with non-normal distributions were expressed as median (interquartile range)

^*^Three female patients on insulin therapy were included twice because they participated twice

^*†*^One of these two patients underwent total pancreatectomy; the other went through Whipple’s operation. Abbreviations: CGM, continuous glucose monitoring; BMI, body mass index; MDI, multiple daily injections; CSII, continuous subcutaneous insulin infusion; eGFR, estimated glomerular filtration rate

The Medtronic’s CGMS Gold ^*TM*^ (Medtronic MiniMed, Northridge, CA, USA) was used to collect continuous glucose data for the durations of 72-96 h with subjects blinded to the data. The CGMS Gold report indicated mean absolute relative difference (MARD) values of 11 to 14%, and a continuous BG error grid plot analysis showed percentage paired values in zone A (optimal) or zone B (acceptable from a clinical/diabetological perspective) to be 98% [29, 30]. BG trends and patterns were identified by retrospective analysis of CGM data. To calibrate the CGM sensors, finger-stick BG levels were measured more than three times per day in all enrolled patients with each measurement made right before each meal. Therefore, the time points at which the BG measurements were recorded for calibration purpose were regarded as the mealtime for this study. The BG measurements within the nocturnal interval (11:00 PM - 7:00 AM) [31] were excluded for the purpose of this study. In addition, if the time interval between two successive BG measurements was less than 2 h, only the last measurement was considered as a meal announcement.

We used Excel (Microsoft, Redmond, USA) to prepare the CGM data and Matlab (Matlab release 2016b, The MathWorks, Natick, 2016) to implement machine learning models and their analyses.

### Data pre-processing and feature extraction

Each CGM time series was presented as a sequence where the *i*^*th*^ CGM time series is given by:

1 $$\begin{array}{@{}rcl@{}}  CGM_{i,:} = \{(CGM_{i,t})\;\; with \;\;t=1,...,N_{i} \} \end{array} $$

where *N*_*i*_ is the length of *CGM*_*i*,:_. For each time series, missing CGM data points were interpolated by the spline method [20] only if less than 3 CGM data points were missing consecutively. The missing CGM data points were reported when the device fails its calibration process [32]. The CGM measurement is taken at every 5 min, and thus *CGM*_*i*,*t*=*n*_ means that the CGM data point at 5 ×*n*^*th*^ minute of the *i*^*th*^ CGM time series. In our study, we took CGM data points after meal announcements and each CGM data point is represented in Eq. 2.

2 $$\begin{array}{@{}rcl@{}}  CGM_{i,j,t} = CGM_{i,meal_{i,j}+t}\;\; with\;\; t \in \{1,...,W\} \end{array} $$

where *meal*_*i*,*j*_ is the time of the *j*^*th*^ meal announcement of the *i*^*th*^ CGM time series, and *W* is the postprandial period.

**Fig. 1:**
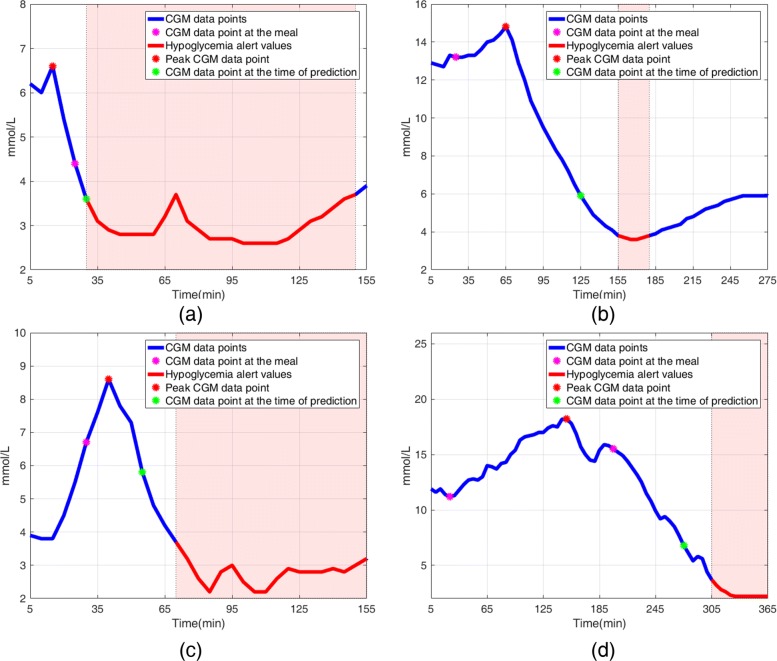
Representative CGM time-series data to show different reactions of selected patients’ glucose levels after meals. Blue line: CGM time-series data points; red line and transparent red box: CGM data point <3.9 mmol/L (70 mg/dL); magenta filled circle: CGM data point at the meal; red filled circle: peak CGM data point after the meal; green filled circle: CGM data point at the time of prediction. Clinical explanations: **a** No peak of CGM data point could occur because the patient ate a small amount of carbohydrates in the meal. **b** Low peak after the meal, then rapid fall in glucose could occur because patient ate a small amount of carbohydrates in the meal. **c** Steep peak, then rapid fall in glucose could occur when the patient ate foods rich in carbohydrate with high glycemic index or injected rapid-acting insulin later than he or she should have. **d** A rapid fall and then no peak after the meal could occur when the insulin injected before the previous meal is still active (insulin on board)

We first analyzed the CGM trends of all selected patients’ data to identify meaningful features for postprandial hypoglycemia. A subset of patients experienced postprandial hypoglycemia if they had a small peak or no peak in CGM, probably due to the meal being small or containing only a small portion of carbohydrate (Fig. 1a and b). There was another group of patients experienced hypoglycemia when the CGM increased steeply and then dropped right after the peak; this reaction probably occurred when the patients ingested carbohydrates with high glycemic index or when the pre-meal rapid-acting insulin was injected too late (Fig. 1c). Insulin injected before a preceding meal can affect a glucose level after the meal. In other cases, a decrease in CGM, in spite of meal ingestion, may have been caused by the insulin on board and was associated with future hypoglycemic episodes (Fig. 1d).

We used above observed data points to define features for predicting hypoglycemia near mealtime. The first feature is defined as ‘the rate of increase in glucose’ (RIG), which is the rate of glucose increase from a meal to a peak:

3 $$\begin{array}{@{}rcl@{}}  RIG_{i,j,t} = \frac{CGM_{i,j,peak_{t}}-CGM_{i,j,0}}{TD_{meal-to-peak}} \end{array} $$

where ${CGM_{i,j,peak_{t}}}$ is the highest CGM data point between the time of the *j*^*th*^ meal announcement of the *i*^*th*^ CGM time series and prediction time *t*, *CGM*_*i*,*j*,0_ is a CGM data point at the *j*^*th*^ meal announcement, and *TD*_*meal*−*to*−*peak*_ is time difference between the meal announcement to the peak. The RIG is updated until the peak CGM data point is found after the meal announcement. If there is no peak CGM data point, the RIG is set to 0. According to the definition of the RIG, zero implies that there is no increase in glucose after the meal.

**Fig. 2:**
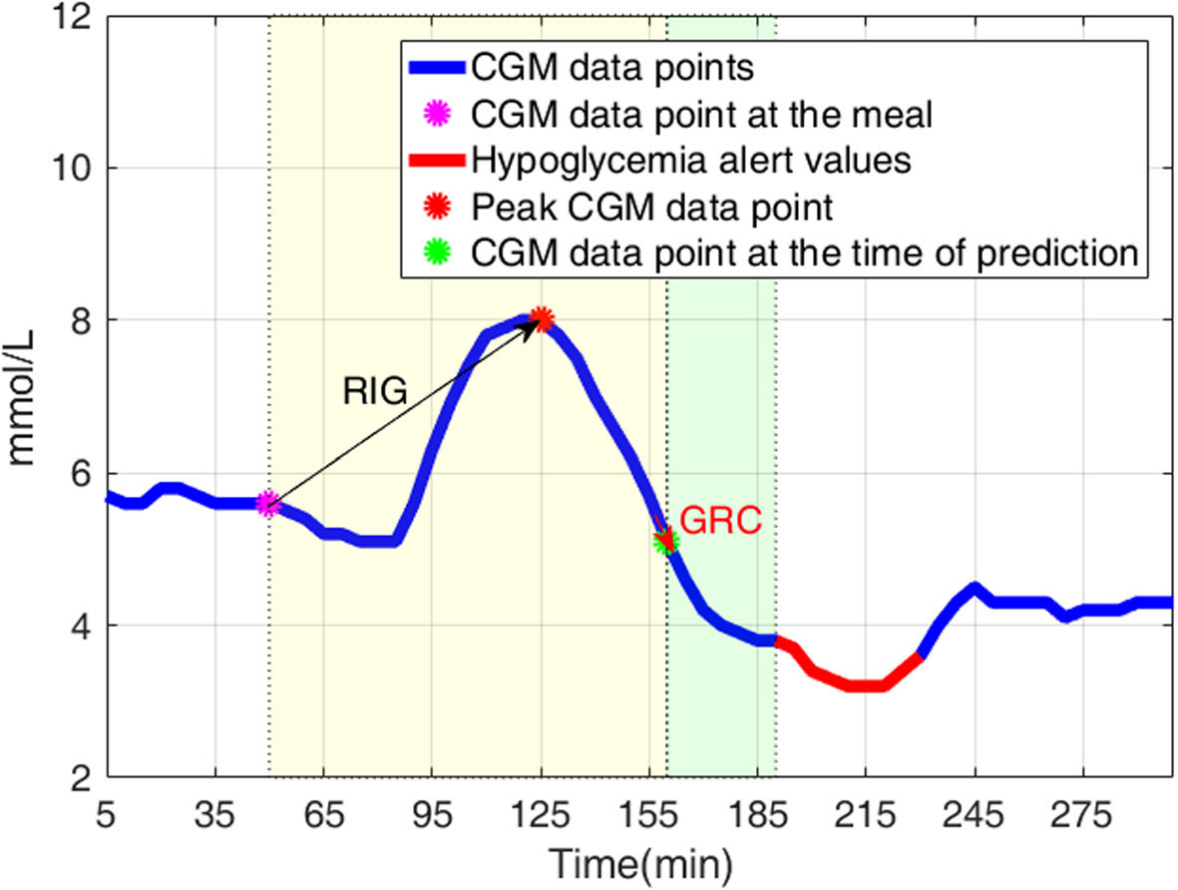
The three features and the 30-min prediction horizon. Blue line: CGM time-series data points; red line: CGM data point <3.9 mmol/L (70 mg/dL); magenta filled circle: CGM data point at the meal; red filled circle: peak CGM data point after the meal; green filled circle: CGM data point at the time of prediction; black arrow: rate of increase in glucose (RIG); red arrow: glucose rate of change (GRC); transparent yellow box: observational window; transparent green box: the 30-min prediction horizon

Since the change in CGM data points is large before hypgolycemia occurs (Fig. 1), we defined the second feature glucose rate of change (GRC) as:

4 $$\begin{array}{@{}rcl@{}}  GRC_{i,j,t} = \frac{CGM_{i,j,t}-CGM_{i,j,t-1}}{5} \end{array} $$

where *CGM*_*i*,*j*,*t*_ is a CGM data point at the time of prediction from the *j*^*th*^ meal announcement of the *i*^*th*^ CGM time series, and *CGM*_*i*,*j*,*t*−1_ is the CGM data point immediately prior to the time of prediction. Since the GRC calculates the near-instantaneous changes in CGM data points around the time of prediction, it can be used to predict hypoglycemia [26, 33]. The third feature is defined to be the CGM data point at the time of prediction (*CGM*_*i*,*j*,*t*_) from the *j*^*th*^ meal announcement of the *i*^*th*^ CGM time series. To define labels, we took into account the presence of a hypoglycemia alert value [34, 35] at the 30-min prediction horizon (i.e., *CGM*_*i*,*j*,*t*+6_). If *CGM*_*i*,*j*,*t*+6_< 3.9 mmol/L (70 mg/dL), we set *Label*_*i*,*j*,*t*_=1. Otherwise, we set *Label*_*i*,*j*,*t*_=0 (Fig. 2).

We obtained all available CGM data points between 5 min and 3.5 h post mealtime announcements (i.e., from *CGM*_*i*,*j*,1_ to *CGM*_*i*,*j*,42_). The corresponding hypoglycemia alert values that occur from 35 min to 4 h after meal announcements were included (i.e., from *Label*_*i*,*j*,1_ to *Label*_*i*,*j*,42_). Although postprandial hypoglycemia can occur later than 4 h after each meal, we chose the window of 35 min to 4 h after the meal because including longer duration after the meal to this time window decreases the prediction accuracy of the algorithm. Since there are already well-established algorithms for predicting fasting or nocturnal hypoglycemia [25, 36], a clinical need of a dedicated algorithm for predicting postprandial hypoglycemia would be most important during the first 4 h after each meal, which is typically difficult to cover using the existing nocturnal hypoglycemic prediction algorithms developed in the setting of gradual changes of blood glucose levels.

The data processing and the feature extraction were performed using the following steps : First, from the *i*^*th*^ CGM time series, the *j*^*t*^*h* meal announcement is selected and the CGM data points from *CGM*_*i*,*j*,1_ to *CGM*_*i*,*j*,42_ were sampled. Second, from the sampled series, *CGM*_*i*,*j*,*t*_,*RIG*_*i*,*j*,*t*_, and *GRC*_*i*,*j*,*t*_ features were extracted while increasing *t* from 1 to 42. The label information is obtained from the CGM data point with the 30-min prediction horizon (i.e., *CGM*_*i*,*j*,*t*+6_).

The first and second steps were repeated for 107 CGM time series around mealtimes, and obtained samples : *D*={(*CGM*_*i*,*j*,*t*_,*RIG*_*i*,*j*,*t*_,*GRC*_*i*,*j*,*t*_,*Label*_*i*,*j*,*t*_) *with* *i*=1,...,107, *j*=1,...,*M*_*i*_, *and* *t*=1,...,42}, where *M*_*i*_ is the total number of meal announcements of the *i*^*th*^ CGM time series. Before training our models, each feature values extracted were normalized with a MinMax Scaler.

### Models

In this study, we selected four commonly used machine learning models with the unique data-driven feature set to predict the occurrence of postprandial hypoglycemia at 30 min from the time of prediction. Four machine learning models were selected [28, 37]: a random forest (RF), a support vector machine using a linear function (SVM-LN) or a radial basis function (SVM-RBF), and a K-nearest neighbor (KNN). Since there are few studies using a logistic regression (LR) [15, 16], we additionally considered the algorithm.

To train and evaluate each model, we used 5-fold cross-subject validation by splitting *D* into *train* *set*_*q*_ and *test* *set*_*q*_ for *q*^*th*^ iteration. The *train* *set*_*q*_ and the *test* *set*_*q*_ were represented as following:

1. *train* *set*_*q*_={(*CGM*_*i*,*j*,*t*_,*RIG*_*i*,*j*,*t*_,*GRC*_*i*,*j*,*t*_,*Label*_*i*,*j*,*t*_) *with* *i*∈*R*_*q*_, *j*=1,...,*M*_*i*_, *t*=1,...,42},*R*_*q*_ is the *q*^*th*^ training folds.
2. $test \; set_{q}=\{{(CGM_{i,j,t},RIG_{i,j,t},GRC_{i,j,t},Label_{i,j,t})}\;with\;i \in (R_{q}^{c}\;\cap \; total\;CGM\; dataset), \;j=1,...,M_{i},\;t=1,...,42\}$.

Data imbalance should be considered before training a model with *train* *set*_*q*_. This imbalance results when the model is mainly trained to predict a majority class and cannot predict a minority class. It is important to predict the minority class since it is the main target in most cases, e.g., postprandial hypoglycemia. For the model to be trained, we chose a false-positive cost and a false-negative cost so that it does not focus on the majority class based on imbalanced data handling [38]. In the training, cost of false negative was determined using Eq. 5 on *train* *set*_*q*_, cost of false-positive was set to 1, true-negative cost and true-positive cost were set to 0.

5 $$ {\begin{aligned}  \mathrm{Cost~of~false~negative} =\frac{\sum\nolimits_{i \in R_{q}}\sum\nolimits_{j=1}^{M_{i}}\sum\nolimits_{t=1}^{42}(1-Label_{i,j,t})}{\sum\nolimits_{i \in R_{q}}\sum\nolimits_{j=1}^{M_{i}}\sum\nolimits_{t=1}^{42}(Label_{i,j,t})} \end{aligned}}  $$

Then, we optimized each model’s hyperparameters with grid search. The best hyperparameters set that resulted in the lowest cross-validation loss, were used for training. During testing, after the *j*^*th*^ meal announcement of the *i*^*th*^ CGM time series, *CGM*_*i*,*j*,*t*_,*RIG*_*i*,*j*,*t*_, and *GRC*_*i*,*j*,*t*_ were extracted at the time of prediction *t*. The features were normalized by the MinMax Scaler trained on a train set. The trained models predict postprandial hypoglycemia with the features. This process was repeated for all *t* increasing from 1 to 42. The number of cases that accurately predicted label vs. inaccurately predicted label were calculated for all meal announcements of the CGM time series in the test set.

**Fig. 3:**
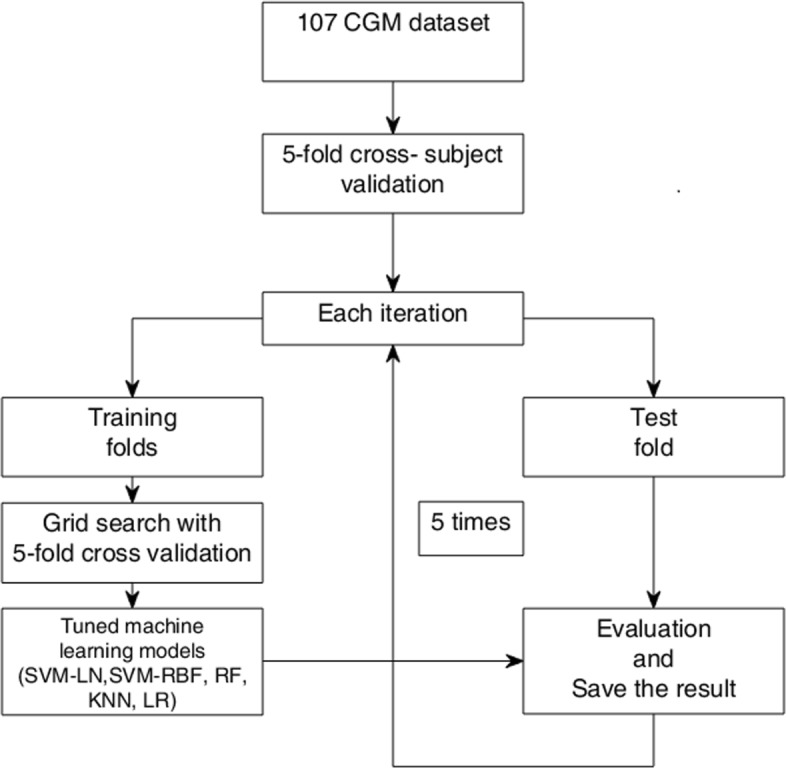
Flowchart of the proposed approach including data-preprocessing, and how to train RF, SVM-LN, SVM-RBF, KNN, and LR. Since the 5-fold cross-subject validation was used, training and testing models were repeated by 5 times. For each iteration, each model’s result and tuned hyper-parameters was saved

The average performance was calculated with the 5-fold cross-subject validation on 107 CGM time series. All training and evaluating process is summarized in the flowchart shown in Fig. 3.

### Metrics

We used four statistical metrics for evaluating the performance of each model: the area under a curve (AUC) of a receiver operating characteristic (ROC) curve, sensitivity, specificity, and F1 score. The AUC for each model is independent of cut-off values on the ROC curve and is mainly used to compare the predictive capabilities of the models, and the AUC is used as the main criterion for indicating the accuracy of the model [39]. Other metrics are defined as follows:

6 $$\begin{array}{@{}rcl@{}}  \mathrm{Sensitivity (or Recall)} = \frac{TP}{TP+FN} \end{array} $$

7 $$\begin{array}{@{}rcl@{}}  \text{Specificity} =\frac{TN}{TN+FP} \end{array} $$

8 $$\begin{array}{@{}rcl@{}}  \text{Precision} =\frac{TP}{TP+FP} \end{array} $$

9 $$\begin{array}{@{}rcl@{}}  \mathrm{F1\ score=2\times\frac{Precision\times Recall}{Precision+Recall}} \end{array} $$

The true positive (*TP*) represents the number of cases predicted correctly to have a hypoglycemia alert value (CGM data point <3.9 mmol/L) at 30 min from the time of prediction and the true negative (*TN*) indicates the number of cases predicted correctly to not have a hypoglycemia alert value at 30 min from the time of prediction. The false positive (*FP*) is the number of cases predicted falsely to have a hypoglycemia alert value at 30 min from the time of prediction, and false negative (*FN*) is the number of cases predicted incorrectly to have a hypoglycemia alert at 30 min from the time of prediction. Sensitivity (or Recall) measures the ability of the algorithm to correctly detect hypoglycemia while specificity measures the ability of the algorithm to correctly detect the absence of it. F1 score is the harmonic mean of both metrics of recall and precision. Since there is a trade-off relationship between precision and recall, F1 score helps to consider both metrics.

To calculate the performance of predictions, the definition of a hypoglycemic event and the definition of an alarm as defined in [19] are used. The main difference between our study and prior study [19] is that we only consider hypoglycemic events in postprandial time interval (5 min to 4 h after a meal). A hypoglycemic event typically has more than two consecutive hypoglycemia alert values. If the time difference between the two events is more than 10 min (two-time steps), the two events are considered as different events. Otherwise, the two events are merged to form the same event. In true alarm, the trained model would predict a hypoglycemia alert value continuously (more than two-time steps) within 60 min before the hypoglycemic event. The detection time (DT) is the time between the first time of an alarm and the first time of a hypoglycemic event. We did not consider alarms within hypoglycemic events as described in [19], but considered only the first alarm within 60 min of each hypoglycemic event.

We calculate the false alarm rate metric, which represents the false alarm ratio among the number of times a patient is alarmed of a hypoglycemic event, as follows:

10 $$\begin{array}{@{}rcl@{}}  \mathrm{False~Alarm~Rate~(FAR)}=\frac{FP_{e}}{TP_{e}+FP_{e}} \end{array} $$

where *TP*_*e*_ is true positive for an event and *FP*_*e*_ is the false positive for an event.

## Results

We analyzed 107 three-day CGM datasets from 104 patients (type-1, *n* = 52; type-2, *n* = 52). In this 107 three-day CGM datasets (99,955 CGM data points at 5-min intervals, the average number of CGM data points per CGM time series was 934), 10.4% had a glucose level <3.9 mmol/L (70 mg/dL). After preprocessing of data, 2062 samples of *D*={(*CGM*_*i*,*j*,*t*_,*RIG*_*i*,*j*,*t*_,*GRC*_*i*,*j*,*t*_,*Label*_*i*,*j*,*t*_) *with* *i*=1,...,107, *j*=1,...,*M*_*i*_, *and* *t*=1,...,42} were included with *Lable*_*i*,*j*,*t*_=1 and remaining were samples with *Lable*_*i*,*j*,*t*_=0. The labels with *Lable*_*i*,*j*,*t*_=0 amounts to about 16.1 times the total number of samples with *Lable*_*i*,*j*,*t*_=1. This difference represents a high data imbalance between the numbers of occurrence of hypoglycemia vs nonoccurrence of hypoglycemia after mealtimes. The total number of postprandial hypoglycemic events within 5 min to 4 h after each meal announcement was 249.

After we trained machine learning models (RF, SVM-LN, SVM-RBF, KNN, and LR) with *train* *set*_*q*_ obtained from *q*^*th*^ training folds, we calculated the statistical performance of each model on each individual *test* *set*_*q*_. Since the 5-fold cross-subject validation was done, 5 performances of each model were used to determine the average performance. The averaged results are summarized in Table 2.

**Table 2:** Average and standard deviation of metrics of models with 5-fold cross-subject validation

Model	Sen (%,SD)	Spe (%,SD)	F1 score (SD)	AUC (SD)	NH (SD)	*TP*_*e*_ (SD)	FAR (SD)	DT (min,SD)
RF	89.6	91.3	0.543	0.966	36.4	30.2	0.704	25.5
	(2.78)	(2.03)	(0.053)	(0.007)	(11.0)	(8.42)	(0.035)	(1.97)
SVM	93.3	88.2	0.487	0.967	36.4	29.2	0.777	25.8
-LN	(1.70)	(2.83)	(0.046)	(0.007)	(11.0)	(8.30)	(0.034)	(2.12)
SVM	89.9	88.8	0.487	0.952	36.4	29.4	0.760	25.2
-RBF	(8.65)	(2.96)	(0.062)	(0.014)	(11.0)	(9.20)	(0.038)	(3.22)
KNN	88.5	89.4	0.492	0.917	36.4	29.6	0.779	25.8
	(1.93)	(2.09)	(0.054)	(0.012)	(11.0)	(8.73)	(0.038)	(3.76)
LR	93.6	87.9	0.484	0.967	36.4	29.6	0.772	25.0
	(2.25)	(2.95)	(0.047)	(0.007)	(11.0)	(8.71)	(0.037)	(2.87)

With the 5-fold cross-subject validation, average metrics were calculated using Eq 6, 7, 9, and 10 on *test* *set*_*q*_,*q*=1,2,3,4,5. Since there should be at least two consecutive predictions of a hypoglycemia alert value to make an alarm, we excluded hypoglycemic events occurring immediately after meals. Abbreviation: RF, random forest; SVM-LN, support vector machine with a linear kernel; SVM-RBF, support vector machine with a radial basis function; KNN, K-nearest neighbor; LR, logistic regression; SD, standard deviation; Sen, sensitivity; Spe, specificity; AUC, the area under the ROC curve; NH, the number of hypoglycemic events; FAR, false alarm rate; DT, detection time.

In order to identify the difference between the average metrics of multiple models, we used statistical analysis methods. The first way is to find models showed the highest metric or the lowest metric, and the second way is to use a one-way analysis of variance (ANOVA) or the Kruskal-Wallis Rank Sum Test for finding a significant difference on the average of a metric of the models. We used the one-way ANOVA only when both normality with the Shapiro-Wilk test and homoscedasticity with Bartlett’s test were satisfied. Otherwise, the Kruskal-Wallis Rank Sum Test was used.

**Fig. 4:**
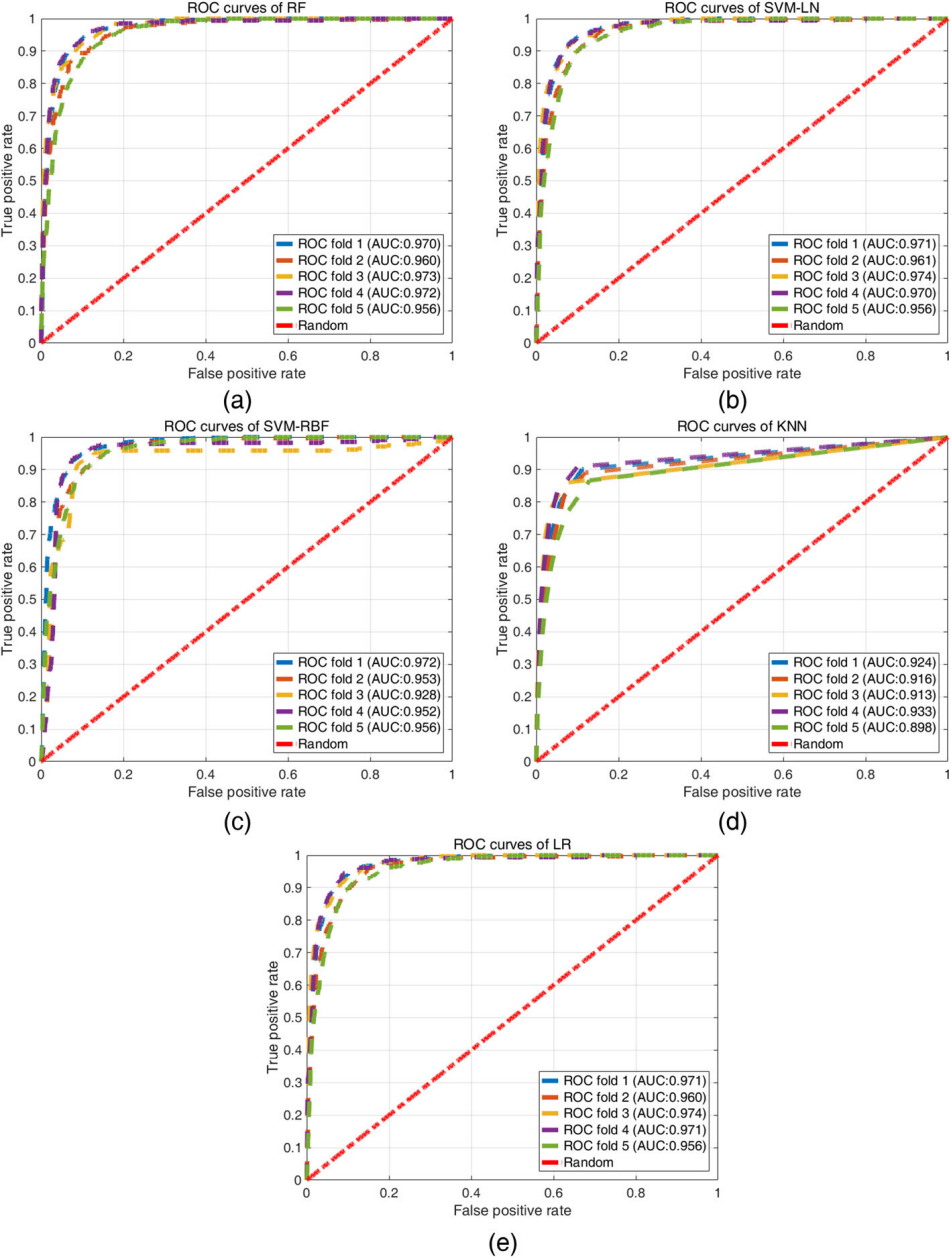
ROC curves for different models. In each iteration of the 5-fold cross-subject validation, the hyper-parameters of the models were determined by the grid search method. **a** ROC curves of RF. **b** ROC curves of SVM-LN. **c** ROC curves of SVM-RBF. **d** ROC curves of KNN. **e** ROC curves of LR. Each colored dashed line represents the ROC curve of each fold. The red dash-dot line indicates a random prediction (i.e., AUC = 0.5)

As shown in Table 2, the average AUC’s of RF, SVM-LN, SVM-RBF, KNN, and LR were 0.966, 0.967, 0.952, 0.917 and 0.967, respectively. All five machine learning models showed high AUC, with LR showing slightly better performance compared to others, implying that the machine learning models have high accuracy and excellent predictive ability [39]. When we used the one-way ANOVA on AUC, there is a significant difference (*p* <0.05). It indicates that KNN shows the worst AUC. Figure 4 shows ROC curves of the different models.

In sensitivity, LR showed the highest average sensitivity (93.6%) and KNN showed the lowest average sensitivity (88.5%). When we used the Kruskal-Wallis Rank Sum Test on sensitivity, there is no significant difference (*p* = 0.06), but the p-value is very close to 0.05. In specificity, RF showed the highest average specificity (91.3%) and LR showed the lowest average specificity (87.9%). In F1 score, RF showed the highest average F1 score (0.543) and LR showed the lowest average F1 score (0.484). In *TP*_*e*_, RF showed the highest average value (30.2) and SVM-LN showed the lowest average value (29.2). In FAR, KNN showed the highest average FAR (0.779) and RF showed the lowest average FAR (0.704). In DT, KNN showed the highest DT (25.8) and LR showed the lowest DT (25.0). With the one-way ANOVA on the metrics including sensitivity, specificity, F1 score, *TP*_*e*_, and DT, there is no significant difference (*p* >0.05). On the other hand, with the one-way ANOVA on FAR, there is a significant difference (*p* = 0.046 <0.05). This indicates that RF is able to be a good model to predict postprandial hypoglycemia.

Since clinically severe hypoglycemia (glucose level less than 3.0 mmol/L, i.e., that is about 54 mg/dL [34]) can lead to catastrophic health issues [35], any predictive model must accurately alarm this clinically significant hypoglycemic events. To calculate the performance of clinically-severe-hypoglycemia alarms, the number of alarmed clinically significant hypoglycemic events was counted for each model. For example, if the alarmed hypoglycemic event has at least one CGM data point <3.0 mmol/L, we regard this event as an alarmed clinically significant hypoglycemic event. On the other hand, if a missed hypoglycemic event has at least one CGM data point <3.0 mmol/L, we consider this event as a missed clinically significant hypoglycemic event. With the 5-fold cross-subject validation, the average number of the hypoglycemic events including at least one CGM data point <3.0 mmol/L was 16.6. The average number of alarmed clinically significant hypoglycemic events made by RF was 14.4 events, by SVM-LN was 14.6 events, by SVM-RBF was 14.2 events, by KNN was 15 events, and LR was 14.4 events. All models alarmed clinically significant hypoglycemic events of more than 86%. With the one-way ANOVA on the alarmed clinically significant hypoglycemic events, there is no significant difference (*p* = 0.989).

To reveal that a part of false alarms were useful, we considered near-hypoglycemic events that includes at least two consecutive CGM data points ≤4.4 mmol/L, i.e., about 80 mg/dL [40]. In other words, we counted all false alarms where near-hypoglycemic events were included within 60 min after the alarms. The 40.0% of average false alarms of RF were related to the near-hypoglycemic events, 28.6% of average false alarms of SVM-LN were related to the near-hypoglycemic events, 31.3% of average false alarms of SVM-RBF were related to the events, 29.9% of average false alarms of KNN were related to the events, and 29.1% of average false alarms of LR were related to the events. With the one-way ANOVA on the percentage, there is a significant difference (*p* <0.05). This result indicates many of false alarms made by the models were related to the near-hypoglycemic events and also indicates that the average false alarms of the RF was not only lowest, but also many RF’s false alarms were associated with the near-hypoglycemic events.

As a result, RF is better in predicting postprandial hypoglycemia with the high level of predictability.

We are the first to use RIG as a new feature. To validate the impact of the feature, we trained another RF considering only two features (i.e., CGM and GRC). As a result, the RF showed 92.2 (4.11) % of the average sensitivity, 89.1 (3.34) % of the average specificity, 0.509 (0.051) of the average F1 score, 0.961 (0.007) of the average AUC, 29.4 (9.39) of the average *TP*_*e*_, 0.742 (0.038) of the average FAR, and 25.7 (2.48) min of the average DT. When we compared the RF trained by all features with the RF trained by two features (i.e., CGM and GRC), there are significant differences in AUC (*p* = 0.033 <0.05) and FAR (*p* = 0.045 <0.05) with Paired t-test. These results establish the importance of RIG in improving the performance.

## Discussion

In this study, the contributions are three folds. First, we verified the feasibility of the RF-based classifier with the simple feature set for predicting postprandial hypoglycemia. In comparing with other commonly used machine learning models, the RF showed the best predictive capabilities with the highest average AUC and superior statistical performance. Second, the proposed methodology uses only a few CGM data points and simple meal announcements. It does not require patients to manually calculate and enter the complex information such as carbohydrate intakes and insulin information. This will minimize patients’ burdens and eventually lower the risk of mistaking data inputs. Third, we found a unique data-driven feature set by intensive review of patient glucose data. The feature set includes the useful RIG (the rate of increase of glucose after a meal) which reflects the steep increase in a glucose level after a meal because of intake of foods with high glycemic index or the late timing of premeal rapid-acting insulin. Moreover, the presence of a postprandial meal peak glucose due to a small amount of meal and a low peak is reflected in RIG. In addition, our study was based on quite large dataset from patients with both types of diabetes (107 CGM cases from 104 patients including 52 people with type 1 diabetes and 52 people with type 2 diabetes), and thus we expect that our proposed method can fit to a general case to predict and prevent postprandial hypoglycemia.

Training models with highly imbalanced dataset is a technically challenging task. This can cause a serious performance distortion. As mentioned in the “Results” section, there was a high imbalance (16.1 times) between hypoglycemia and non-hypoglycemia. This data imbalance problem can be solved using approaches [41] such as over/under sampling, cost-based learning, etc. Among these approaches, we used the cost-sensitive learning to utilize full data samples and avoid training with redundant data samples. We have assigned different costs between FP and TN to solve the problem of highly imbalanced dataset, and have trained the models to predict more hypoglycemia. As a result, the five models showed the high sensitivity greater than 88%, big *TP*_*e*_, and the large number of alarmed events including at least one CGM data point <3.0 mmol/L (about 54 mg/dL), which is considered as clinically significant hypoglycemia. Although these results have showed high FAR, many false alarms have been found to be associated with near-hypoglycemic events that have two consecutive CGM data points ≤4.4 mmol/L (about 80 mg/dL). This means that there is a lot of glucose fluctuations around mealtimes. Unlike nocturnal hypoglycemia, it is because there may be unpredictable interventions from people with diabetes in the daytime. For example, the patients may be exercising, stressed, or taking sugary drinks or snacks. Accurate predictions of postprandial may require more user unfriendly manual inputs, but this increases the burden on the patient and the chance of users’ mistakes in entering information. Thus, it is necessary to develop a system that can automatically process the patient’s lifestyle data, or more studies, which predict the occurrence of postprandial hypoglycemia with only CGM measurements and easy input, are needed.

Our results showed that the ensemble way that uses multiple single learners to make a decision with a voting has the better predictability than the single model such as SVM-LN, SVM-RBF, KNN, and LR. This implies that the ensemble approach has better generalization capabilities compared to other models on predictions of the occurrence of postprandial hypoglycemia during various glycemic changes which are affected by carbohydrate in a meal and injected insulin doses. Thus, we selected RF as our primary model to predict the occurrence of postprandial hypoglycemia, and other model were used to contrast the performance. For future advanced studies, the process that optimizes the structure of an ensemble method such as stacking multiple models, soft voting, and hard voting and selects appropriate machine learning models is needed. Furthermore, it is also necessary to take into account the computational complexity of the ensemble model for working on a compact device. Although this process may require complex procedures and lots of time, it is expected that it will enable the development of a model predicting more accurately the occurrence of postprandial hypoglycemia without any manual inputs.

For patients, the extended prediction horizon is beneficial because it increases the time available for a patient to take action to prevent potential hypoglycemia. However, it should be noted that there is a trade-off relationship between the prediction horizon and the accuracy of a model [42]. Generally, increasing the prediction horizon will lower the accuracy and priority should be decided based on clinical needs. For example, patients who want to know the occurrence of hypoglycemia earlier, in spite of many false alarms, will prefer a long-term prediction horizon. Conversely, a short-term prediction horizon will be preferred for patients who want to know the occurrence of hypoglycemia with higher confidence. The primary reason for choosing the 30-min prediction horizon was the good trade-off between the prediction horizon and the accuracy of prediction [37]. The 30-min prediction horizon enabled an acceptable accuracy while providing an effective time for correcting hypoglycemia with carbohydrate ingestion or injection of glucagon. In addition, several studies have used the 30-min prediction horizon [17–21, 23, 37, 43] and have verified that this time is sufficient to prevent hypoglycemia in patients [17]. Therefore, we believe the 30-min prediction horizon used in this study would be adequate for alarming people with type 1 diabetes to take carbohydrate or for alarming a bihormonal AP system to infuse glucagon, but the 30-min prediction horizon might be inadequate for prevention of hypoglycemia only by reduction of insulin infusion rate in single hormone AP system.

Besides AP system, the model is also useful for stand-alone real-time CGM device since it requires only mealtime announcement and CGM data for its operation. Meal announcement is manual but can easily performed by pressing a button on the device. In many type-1 or insulin-treated type-2 diabetes patients, who cannot use an insulin pump, multiple daily injection insulin therapy with real-time CGM is a reasonable option [44]. Widespread use of flash BG monitoring, which can replace a finger-stick glucometer even for insulin-treated type-2 diabetes patients. In these clinical settings, the feature of our algorithm that does not require insulin dosing information could be a benefit for patients who do not use insulin pumps [45].

To collect three-day CGM data points from 104 people with type 1 and type 2 diabetes, Medtronic’s CGMS Gold ^*TM*^ was used. This device retrospectively calibrated and filtered collected CGM data points at the end of the monitoring. Thus, the collected CGM traces are smoother than the real-time CGM traces.

It is important to acknowledge the limitations of the study. First, tests were performed retrospectively with 107 three-day CGM datasets, and a prospective study should be conducted to assess the clinical credibility of the prediction algorithm. Second, 30-minute, which was the prediction horizon, may be not an enough time to avoid hypoglycemia without the ingestion of carbohydrates or injection of glucagon. Last, a patient should announce mealtimes to operate our algorithm. Although it adds a manual activity to the patient, it is still a much less burdensome activity than counting carbohydrates and entering injected insulin dose that other algorithms require. As the next step, we will develop a meal detection algorithm by using CGM data and accumulated patients’ mealtime information. It is expected to greatly improve the usability of the hypoglycemia prediction algorithm.

## Conclusions

In this study, we could successfully identify hypoglycemia using the RF-based model in the postprandial situation. The algorithm could predict a hypoglycemia alert value in a clinically-useful 30-min prediction horizon around mealtimes. This proposed approach only requires CGM data points and simple mealtime announcements, and is less burdensome to patients than models using lots of input information. This study not only establishes a new methodology to predict postprandial hypoglycemia but also verifies the feasibility of RF to accurately predict postprandial hypoglycemia. We believe that the proposed machine learning approach can be integrated with real-time CGM devices and sensor-based AP system, so it will be a great help for people with diabetes to manage their glucose level and improve their quality of life. In the near future, we will evaluate our algorithm on a prospective patient population to clearly establish the clinical use of this system.

## Data Availability

The data that support the findings of this study are available from Samsung Medical Center but restrictions apply to the availability of these data. The data were used under license for the current study, and so are not publicly available. Data are however available from the authors upon reasonable request and with permission of Samsung Medical Center.

## Abbreviations

AP: Artificial pancreas
CGM: Continuous glucose monitoring
CSII: Continuous subcutaneous insulin infusion
FAR: False alarm rate
FGM: Flash glucose monitoring
KNN: K-nearest neighbor
MDI: Multiple daily injection
RF: Random forest
SVM-LN: Support vector machine with a linear kernel
SVM-RBF: Support vector machine with a radial basis function
